# Mouse adaptation of H6 avian influenza viruses and their molecular characteristics

**DOI:** 10.3389/fmicb.2022.1049979

**Published:** 2022-11-17

**Authors:** Zhimin Wan, Jianxi Gong, Jianjun Sang, Wenjie Jiang, Zhehong Zhao, Mingjun Lian, Ting Tang, Yafeng Li, Qiuqi Kan, Quan Xie, Tuofan Li, Hongxia Shao, Wei Gao, Aijian Qin, Jianqiang Ye

**Affiliations:** ^1^Key Laboratory of Jiangsu Preventive Veterinary Medicine, Key Laboratory for Avian Preventive Medicine, College of Veterinary Medicine, Ministry of Education, Yangzhou University, Yangzhou, Jiangsu, China; ^2^Jiangsu Co-innovation Center for Prevention and Control of Important Animal Infectious Diseases and Zoonoses, Yangzhou, Jiangsu, China; ^3^Joint International Research Laboratory of Agriculture and Agri-Product Safety, Ministry of Education of China, Yangzhou University, Yangzhou, Jiangsu, China; ^4^Institute of Agricultural Science and Technology Development, Yangzhou University, Yangzhou, Jiangsu, China; ^5^Sinopharm Yangzhou VAC Biological Engineering Co. Ltd, Yangzhou, Jiangsu, China

**Keywords:** H6 AIV, mouse adaptation, amino acid substitutions, pathogenicity, molecular mechanism, receptor binding, polymerase activity

## Abstract

H6 avian influenza viruses (AIVs) not only continue to circulate in both domestic poultry and wild waterfowl, but also have occasionally caused spillovers infections in pigs and humans, posing a potential threat to public health. However, the molecular mechanism of H6 AIV adaptation to mammals remains largely unknown. In this study, two mouse-adapted (MA) H6 AIV strains, named as MA E-Teal/417 and MA GWF-Goose/740, were generated through blind passages in BALB/c mice. The two MA H6 strains replicated more efficiently and showed higher virulence than the corresponding wild type (WT) H6 strains in mice. Genome sequencing revealed that MA E-Teal/417 and MA GWF-Goose/740 carried six amino acid mutations (PB2-T224A/E627K, HA-G124R, NA-F167L/Y356H and M1-M92R), and four amino acid mutations (PB1-K577E, PA-T97I/D514E and HA-T276K), respectively, when compared to the corresponding WT virus. Receptor binding assay showed MA E-Teal/417 had stronger binding activity to α-2,3 SA than WT E-Teal/417. Moreover, the polymerase activity analysis found the RNP polymerase activity of both MA H6 viruses was significantly higher than that of the corresponding WT virus in 293T cells. All these demonstrate that H6 AIV can acquire limit amino acid substitutions to adapt to mammals and increase virulence, highlighting the significance of monitoring such mutations of H6 AIV in the field for alarming the potential of its cross-transmission and pathogenesis in mammals.

## Introduction

Avian influenza viruses (AIVs) belong to the *Orthomyxoviridae* family and contain eight single-stranded negative RNA segments, which encode 10 essential proteins and several accessory proteins. Based on the antigenic properties of two surface glycoproteins, hemagglutinin (HA) and neuraminidase (NA), AIVs were subgrouped into 16 HA and 9 NA subtypes (Tong et al., 2013; Araujo et al., 2018). Migratory waterfowl are the nature reservoirs for AIVs (Webster et al., 1992), which constantly cross barrier and jump into other hosts, such as poultry and mammals, including human (Liu et al., 2003; Capua and Alexander, 2004; Mostafa et al., 2018). Although the threat of AIVs posed by H5 and H7 subtypes is most prominent, the risks of other subtype AIVs cannot be ignorable, one of which is H6 AIV.

H6 AIV was first isolated from turkeys in United States in 1965 (Downie and Laver, 1973). Currently, H6 AIVs have been identified in various animal species in different countries (Munster et al., 2007), and contributed to the genomic exchange and diversity of AIVs in wild birds (Woolcock et al., 2003; Kim et al., 2010; Fuller et al., 2015). Since 2002, H6 AIV has been one of the predominant AIV subtypes circulating in live bird markets in southern China and more than 30% H6 AIVs circulating in poultry in China increase the affinity to human-like receptor (Huang et al., 2010; Wang et al., 2014). On 2013, the first case of a human infection with an avian-origin H6N1 was report in Taiwan (Wei et al., 2013; de Vries et al., 2017), and some poultry workers showed the serum positive for the H6 in China (Wu et al., 2015). Avian-origin H6N6 AIVs were isolated from sick pigs in China from 2010 (Zhao et al., 2013; Sun et al., 2017). In addition, H6 AIVs have been shown to replicate efficiently in mice without adaptation (Ge et al., 2017; Cui et al., 2021; Li et al., 2021; Wan et al., 2021). All these events demonstrate that H6 AIVs can cause cross-species infection in mammals. However, the molecular mechanism of the adaptation of H6 AIV in mammals remains to be elucidated.

The mouse model is widely used to study the pathogenesis and adaptation of AIVs infection in mammals. The mouse-adapted (MA) AIV strains can be generated by serial lung-to-lung passaging, which is commonly used to study the critical genes and/or amino acid sites associated with cross-species infections of AIVs in mammals (e.g., H1, H3, H4, H5, H9, and H10 AIVs; Keleta et al., 2008; Sang et al., 2015; Yu et al., 2017; Zhang et al., 2017a,b; Xu et al., 2020). In this study, five H6 AIVs isolated from wild birds were adapted in BALB/c mice, and two MA H6 viruses were efficiently generated and characterized.

## Materials and methods

### Cells and viruses

Human kidney cell (293T), Madin–Darby canine kidney cell (MDCK) and human lung pulmonary epithelial cell (A549) were grown in Dulbecco’s modified Eagle’s medium (DMEM; Gibco, United States) supplemented with 10% fetal bovine serum (FBS) and 1% antibiotic. These cells were propagated at 37°C in 5% CO_2_. A/Eurasian teal/Jiangxi/2018WB0049/2018 (H6N2; E-Teal/49), A/Eurasian Wigeon/Jiangxi/2018 WB0158/2018 (H6N2; E-Wigeon/158), A/Eurasian Wigeon/Jiangxi/2018WB0266/2018(H6N2; E-Wigeon/266), A/Eurasian teal/Jiangxi/2018WB0417/2018(H6N2; E-Teal/417), and A/Greater White-fronted goose/Jiangxi/2018WB0740/2018(H6N1; GWF-Goose/740) were isolated from wild birds in Poyang Lake in China (Wan et al., 2021). These viruses were amplified in 10-day-old specific-pathogen-free (SPF) embryonated chicken eggs and stored at –80°C.

### Adaptation of H6 AIVs in mouse

To obtain the MA H6 AIVs, the five H6 AIVs from wild birds adapted in mice through serial passages. Briefly, 15 5-week-old BALB/c mice were randomly divided into 5 groups (3 mice/group). The mice were anesthetized with isoflurane and infected with each H6 AIV at 10^5^ TCID_50_ virus in 25 μl of PBS by intranasal inoculation. At 5 day post-infection (dpi), the mice of each group were sacrificed and the lungs were collected, homogenized and centrifuged at 5000 *g* for 10 min, and the 25 μl of the supernatant was used to infect mouse for the next passage. After serial passages, the MA H6 viruses present in the lung homogenate were cloned by plaque purification in MDCK cells, and then were amplified in 10-day-old SPF embryonated chicken eggs.

### Viral growth kinetics *in vitro*

Confluent MDCK or A549 cells were infected with each MA and WT H6 AIV at a multiplicity of infection (MOI) of 0.01, and maintained in opti-MEM media containing 1 μg/ml TPCK-trypsin and incubated at 37°C in 5% CO_2_. The tissue culture supernatants were harvested at 6, 12, 24, 48, and 72 h post-infection (hpi), and then viral titers were measured by TCID_50_ in MDCK or A549 cells. The experiments were performed in triplicates.

### Pathogenicity assay in mouse

To determine the virulence of MA H6 viruses in mice, 5-week-old BALB/c mice were anesthetized with isoflurane and infected with MA or WT H6 viruses at 10^5^ TCID_50_ virus in 25 μl of PBS by intranasal inoculation. The clinical signs, body weight loss and mortality of the infected mice were monitored for 14 days. Mice with bodyweight loss of more than 25% were humanely euthanized. At 3 and 5 dpi, three mice of each group were euthanized and the lung, brain, liver and kidney samples were collected for viral titration. Notably, the lungs collected at 5 dpi were also used for histopathology analysis. To further determine the 50% mouse lethal dose (MLD50) of MA H6 AIVs, 5-week-old BALB/c mice were anesthetized with isoflurane and infected intranasally with 10-fold serial dilutions of MA H6 viruses. The clinical signs, bodyweight loss and mortality of the infected mice were monitored for 14 days. Mice with weight loss of more than 25% were humanely euthanized. The MLD_50_ was calculated and expressed as TCID_50_.

### Sequencing analysis

Viral RNAs were extracted using RNA isolation kit (Vazyme Biotech Co., Ltd.). Two-step reverse transcription PCR was performed using specific primers for each gene segment as previously described (Shao et al., 2015), and the products were sequenced by Sanger sequencing. Nucleotide and deduced amino acid sequences were compiled and aligned using the DNAStar (v7.1).

### Receptor binding assay

The receptor binding property of these H6 viruses was analyzed by solid-phase binding assay as previously described (Liu et al., 2018). Briefly, the Perce streptavidin high binding capacity coated 96-well plates (Thermo, Rockford, United States) were coated with serially 2-fold diluted Neu5Aca2-3Galb1-4GlcNAcb-PAA-biotin or Neu5Aca2-6Galb1-4GlcNAcb-PAA-biotin (Glycotech, Gaithersburg, MD, United States) and incubated overnight at 4°C. After blocked with 5% skimming milk in PBST, the plates were incubated at 4°C overnight with 64 HAUs of the H6 viruses. After washed three times with PBST, the plates were incubated with H6-specific murine monoclonal antibody 5G2. The plates were washed three times with PBST and incubated with a horseradish peroxidase (HRP)-conjugated anti-mouse IgG antibody for 2 h at 4°C and then washed three times with PBST again. Then 100 μl TMB solution was added to each well and incubated 10 min. The reaction was stopped by adding 2M H_2_SO_4_, and the OD_450_ values were measured.

### Luciferase assay for polymerase activity

Polymerase activity was measured using a mini-genome assay as previously described (Song et al., 2011). Briefly, 293 T cells were co-transfected with a luciferase reporter plasmid p-Luci together with internal control Renilla plasmid and RNP genes (PDP2002-PB2, PDP2002-PB1, PDP2002-PA, and PDP2002-NP) either from MA H6 or from WT H6, and incubated for 48 h. The luciferase activity was measured using a dual-luciferase reporter system (Vazyme Biotech Co., Ltd.) according to the manufacturer’s instructions.

### Statistical analysis

All the results are presented as means ± standard deviations. The statistical analysis in this study was performed with a Student *t* test using Graphpad 5 software. A *p* value of below 0.05 was considered significant. ^*^, ^**^, and ^***^ indicate *p* values of less than 0.05, 0.01, and 0.001, respectively.

## Results

### Generation of two MA H6 AIVs

**Figure 1:**
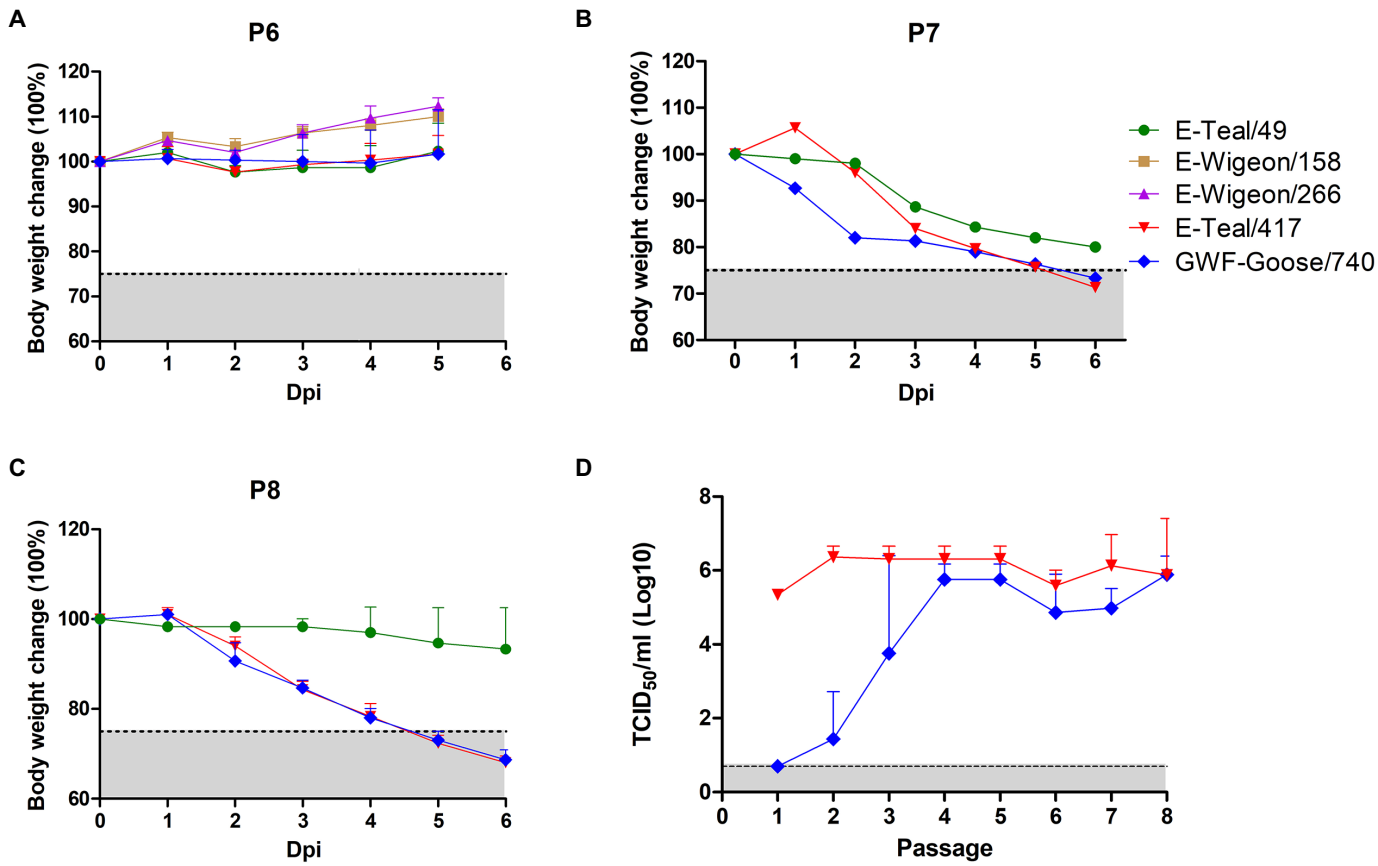
Adaptation of H6 AIVs in mice. 5-week-old BALB/c mice were inoculated with 10^5^ TCID_50_ of each H6 virus, then the viruses in lung homogenates were passaged in BALB/c mice. The body weight change of infected mice was recorded in P6 **(A)**, P7 **(B),** and P8 **(C)**. **(D)** The virus titers in lung homogenates of E-Teal/417 and GWF-Goose/740 from each passage were measured by TCID_50_ in MDCK cells each passage.

Our previous study has demonstrated that all the five H6 AIVs (E-Teal/417, E-Wigeon/158, E-Wigeon/266, E-Teal/417, and GWF-Goose/740) were low pathogenic for mice, whereas two of them (E-Teal/49 and E-Teal/417) could replicate in the lung of mice and the other three (E-Teal/158, E-Teal/266, and GWF-Goose/740) could not (Wan et al., 2021). To generate the MA H6 AIVs and elucidate the molecular determiners for the mouse adaptation, the five H6 AIVs were adapted in mice by serially passaging the lung homogenates from the infected mice. Compared with the mice infected with other H6 AIVs, some mice infected with E-Teal/49, E-Teal/417, and GWF-Goose/740 began to lose bodyweight at 6^th^ passage (P6) of adaptation. Therefore, E-Teal/49, E-Teal/417 and GWF-Goose/740 were selected to continue to be passaged. All the mice infected with E-Teal/417 and GWF-Goose/740 showed bodyweight loss beyond 25% and had to be euthanized at 6 dpi in P8 (Figures 1A–C). At the same time, the viral load in lung of mice infected with E-Teal/417 and GWF-Goose/740 were titrated for each generation. As described in Figure 1D, the average viral titers of GWF-Goose/740 in lung gradually increased after lung-to-lung passaging, and those of E-Teal/417 remained high in each passage. These results indicated that E-Teal/417 and GWF-Goose/740 had acquired adaptive mutations that significantly increased virulence in mice. The E-Teal/417 and GWF-Goose/740 viruses of P8 were plaque-purified in MDCK cells and amplified in 10-day old SPF eggs, designated as MA E-Teal/417 and MA GWF-Goose/740.

### Growth kinetics of the MA H6 viruses in MDCK and A549 cells

**Figure 2:**
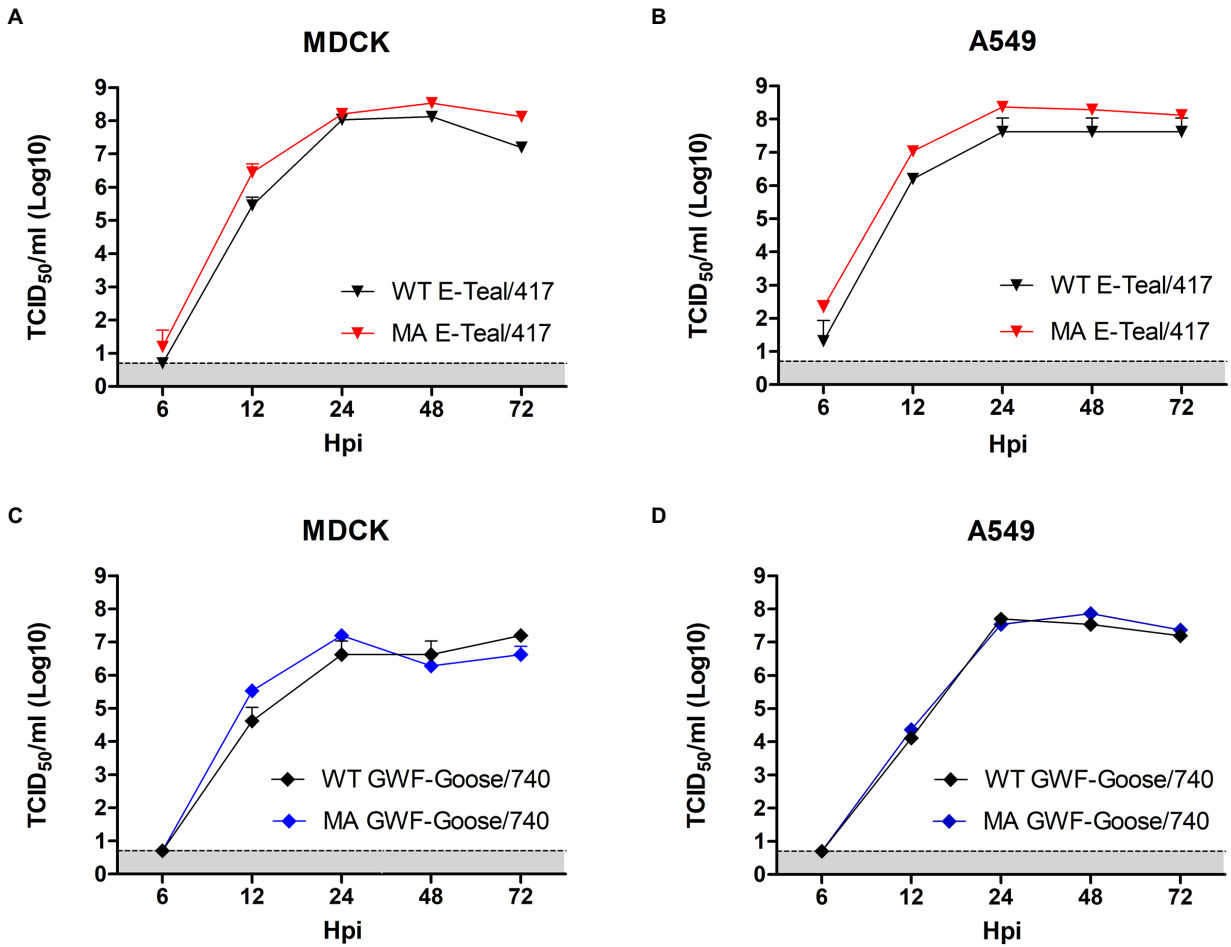
Growth kinetics of the MA and WT H6 AIVs in MDCK **(A,C)** and A549 **(B,D)** cells. Confluent MDCK or A549 cells were infected with each virus at an MOI of 0.01. Supernatant samples were collected at 6, 12, 24, 48, and 72 hpi, and viral titers were measured in MDCK or A549 cells by TCID_50._

To compare the viral replication ability of the MA H6 viruses with the corresponding WT viruses *in vitro*, the viral growth kinetics of both MA and WT H6 viruses in MDCK and A549 cells were carried out. As shown in Figure 2, all these viruses replicated efficiently in both cell lines. MA E-Teal/417 grew to higher titers than that of WT E-Teal/417 throughout the whole time course in both cell lines, and peaked at 24 hpi with titers of about 10^8^ TCID_50_/ml (Figures 2A,B). Compared with WT GWF-Goose/740, MA GWF-Goose/740 grew faster in MDCK cells, and peak at 24 hpi with titer of about 10^7^ TCID_50_/ml, and the two viruses showed similar replication kinetics in A549 cells (Figures 2C,D). Notably, the peak titers of MA E-Teal/417 and WT E-Teal/417 were 10-fold higher than those of MA GWF-Goose/740 and WT GWF-Goose/740. All these demonstrate that although WT E-Teal/417 and WT GWF-Goose/740 can efficiently replicate in both MDCK and A549 cells without pre-adaptation in mammal species, the MA E-Teal/417 does show higher replication efficiency in both MDCK and A549 cells.

### Pathogenicity of the MA H6 viruses in mouse

**Figure 3:**
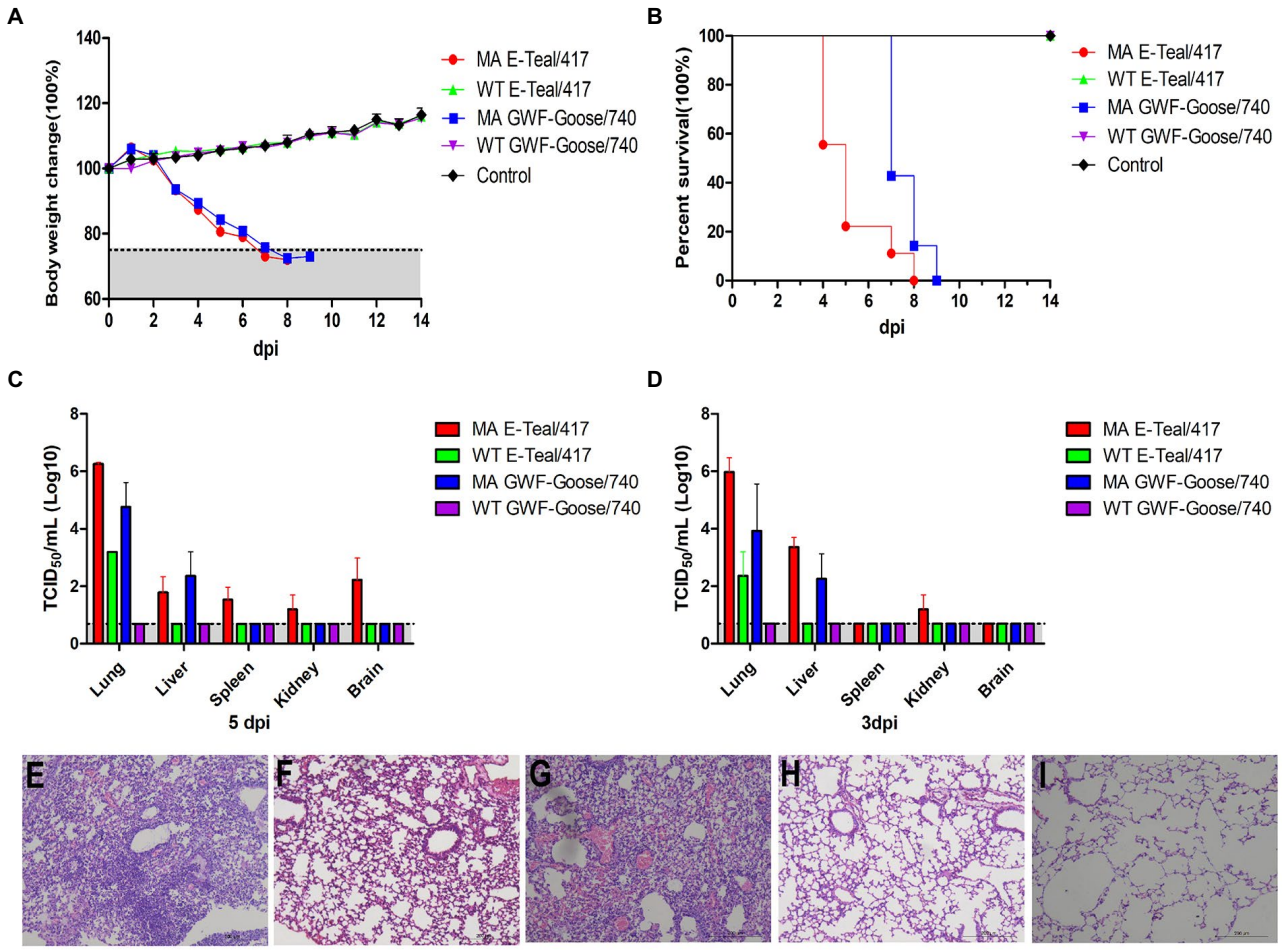
Pathogenicity of the MA H6 AIVs in mice. 5-week-old BALB/c mice were infected intranasally with 10^5^ TCID_50_ of each MA and WT H6 virus. Percentages of change in body weight and survival following infection with each virus **(A,B)**. The viral titers of lungs, livers, spleens, kidneys, and brains collected at 3 and 5 dpi were measured in MDCK cells **(C,D)**. Lungs collected at 5 dpi were fixed in 10% formalin, embedded in paraffin and sectioned. Serial section were stained with H&E. **(E)** MA E-Teal/417, **(F)** WT E/Teal/417, **(G)** MA GWF-Goose/740, **(H)** WT GWF-Goose/740, and **(I)** Mock.

To further investigate the pathogenicity of the MA H6 viruses, 5 -week-old BALB/c mice were infected intranasally with 10^5^ TCID_50_ of MA E-Teal/417, MA GWF-Goose/740, WT E-Teal/417, and WT GWF-Goose/740, respectively. Clinical signs of disease, bodyweight change and mortality of the infected mice were recorded daily. The mice infected with MA E-Teal/417 and MA GWF-Goose/740 showed rapid bodyweight loss after infection, and succumbed or had to be euthanized at 8 and 9 dpi as described in Figures 3A,B. As expected, the mice infected with the corresponding WT viruses did not show any bodyweight loss. Three mice of each group were euthanized and the lung, liver, spleen, and brain samples were collected to determine the virus load at 3 and 5 dpi, respectively. As shown in Figures 3C,D, the two MA H6 viruses showed significantly higher viral titers in lungs than the WT H6 viruses at 3 and 5 dpi. Moreover, both MA E-Teal/417 and MA GWF-Goose/740 could replicate in liver. Notably, expect for lung and liver, MA E-Teal/417 could be detected in spleen and kidney at 3 dpi and in spleen, kidney and brain at 5 dpi. In addition, the lung histopathology analysis revealed that WT E-Teal/417 and WT GWF-Goose/740 did not caused any lesions, whereas MA E-Teal/417 and MA GWF-Goose/740 induced sever pneumonia, characterized by the infiltration of inflammatory cells and hemorrhage (Figures 3E–I).

**Figure 4:**
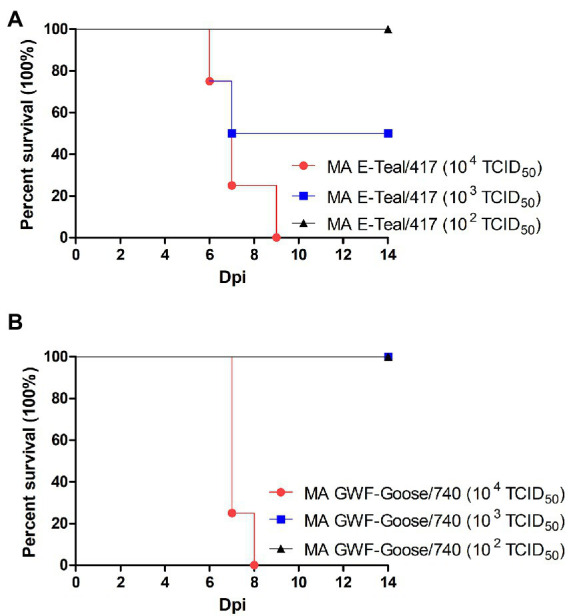
MLD_50_ of the two MA H6 AIVs. 5-week-old BALB/c mice (4 mice/group) were inoculated intranasally with different doses of virus (10^2^–10^4^ TCID_50_ of MA Teal/417 and MA GWF-Goose/740). Survival following infection with **(A)** MA E-Teal/417 and **(B)** MA GWF-Goose/740 were recorded.

To compare the virulence of the two MA H6 viruses, the MLD_50_ of MA E-Teal/7 and MA GWF-Goose/740 was titrated in 5 -week-old BALB/c mice. As described in Figure 4, all the mice infected with 10^4^ TCID_50_ MA E-Teal/417 and MA GWF-Goose/740 were died or had to be sacrificed, and mice infected with 10^3^ TCID_50_ MA E-Teal/417 caused 50% mortality. The other infected mice were all survived. After calculation, the MLD_50_ of MA E-Teal/417 and MA GWF-Goose/740 were 10^3^ TCID_50_ and 10^3.5^ TCID_50_, respectively. All these demonstrate that MA E-Teal/417 and MA GWF-Goose/740 are highly pathogenic to BALB/c mouse after adaptation.

### Molecular determiners in the MA H6 viruses

To identify the virulence-associated molecular basis of MA E-Teal/417 and MA GWF-Goose/740, the full genomes of the two MA H6 and the corresponding WT viruses were sequenced. In comparison with the corresponding WT viruses, sequence data revealed six amino acid substitutions, PB2-T224A/E627K, HA-G124R, NA-G167P/Y256H, and M1-M192R, were found in MA E-Teal/417; and four amino acid substitutions, PB1-K577E, PA-T97I/D514E, and HA-T276K, were present in MA GWF-Goose/740 (Tables 1, 2). These mutations found in MA E-Teal/417 and MA GWF-Goose/740 might be important for the mouse adaptation of WT E-Teal/417 and WT GWF-Goose/740 AIVs. To further analyze whether the amino acid mutations found in the two MA H6 viruses have occurred in the field, all H6 sequences deposited in Influenza Research Database^1^ were analyzed. As described in Tables 1, 2, PB2-T224A/E627K, HA-G124R, NA-Y356H in MA E-Teal/417, and PA-T97I, HA-T276K in MA GWF-Goose/740, were already present in some of field H6 isolates, indicating some of the field H6 AIVs have acquired amino acid substitutions, which might help them adapt to mammals and increase the pathogenicity.

**Table 1:** Amino acid substitutions in the MA E-Teal/417 and their distribution in H6 AIVs.

Proteins	AA^a^ substitutions	H6 subtype
PB2	T224A	T (1745/1746), A(1/1746)
	E627K	E(1738/1746), K(6/1746)
HA	G124R	G(2013/2256), R(28/2256)
NA	F167L	F(821/821), L(0/821)
	Y356H	Y(247/821), H(3/821)
M1	M192R	M(2,120/2131), R(0/2131)

a AA, amino acid.

**Table 2:** Amino acid substitutions in the MA GWF-Goose/740 and their distribution in H6 AIVs.

Proteins	AA^a^ substitutions	H6 subtype
PB1	K577E	K(1798/1845), E(0/1845)
PA	T97I	T(1780/1808), I(12/1808)
	D514E	D(1807/1808), E(0/1808)
HA	T276K	T(1,333/2256), K(569/2256)

a AA, amino acid.

### Receptor binding specificity of MA H6 viruses

**Figure 5:**
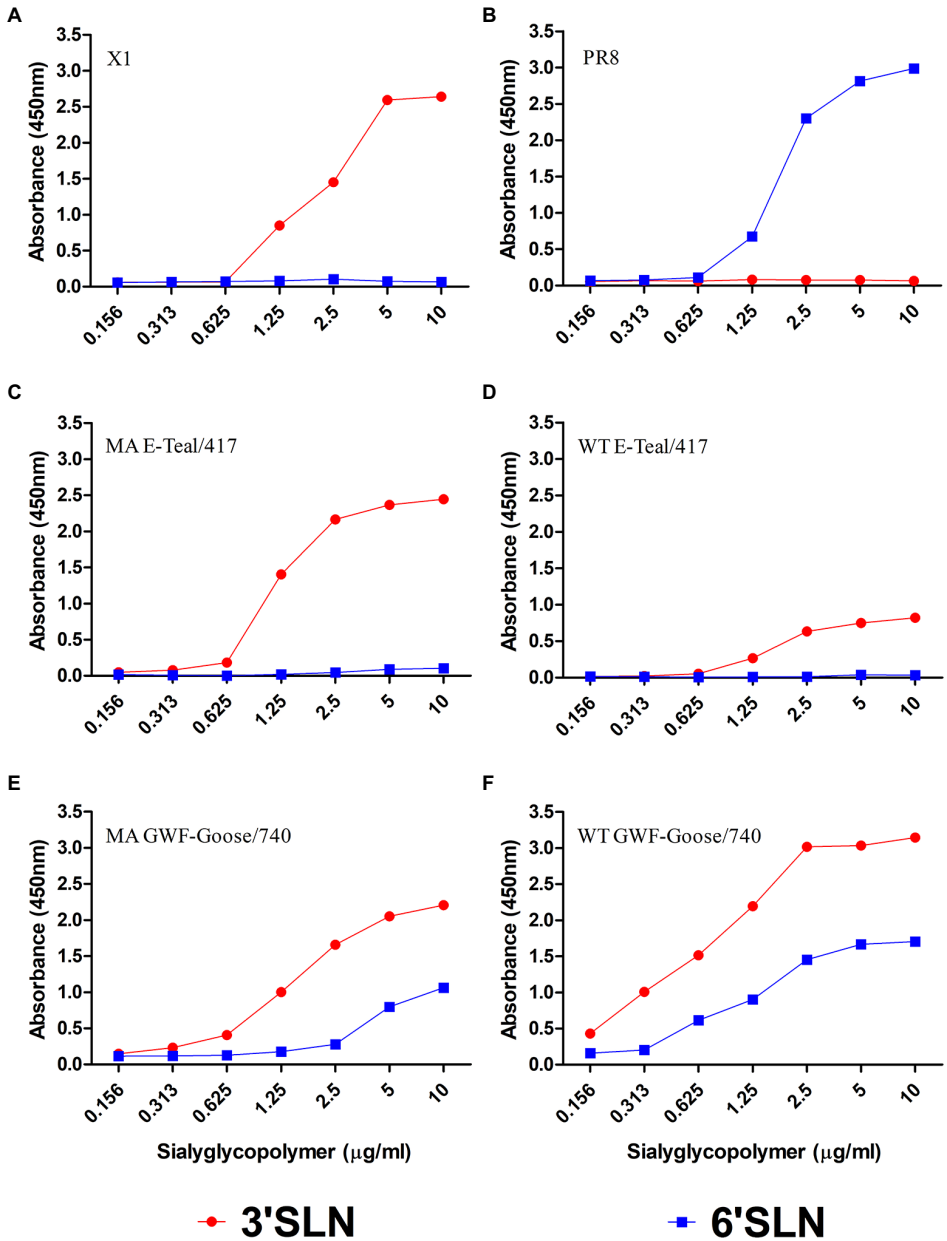
Receptor-binding properties of the two MA H6 viruses. The binding of **(C)** MA E-Teal 417, **(D)** WT E-Teal/417, **(E)** MA GWF-Goose/740, and **(F)** WT GWF-Goose/740 with sialic acids were determined using various concentrations of sialic acids conjugated to biotinylated sialylglycopolymers (3′SLN and 6′SLN) *via* direct solid-phase binding assays. **(A)** X1 and **(B)** PR8 were the controls for α-2, 3 SA and α-2, 6 controls.

Receptor binding specificity plays critical roles in the transmission and host adaptation of influenza virus between avian and mammals. Compared with WT viruses, MA E-Teal/417 and MA GWF-Goose/740 have a mutation in HA (G124R in MA E-Teal/417 and T276K in GWF-Goose/740), respectively. To assess whether G124R in MA E-Teal/417 and T276K in GWF-Goose/740 affect the receptor binding specificity, the MA and WT H6 viruses were tested with two receptor analogs, i.e., Neu5Acα2-3Galb1-4GlcNAcb (3′SLN) and Neu5Acα2-6Galb1-4GlcNAcb (6′SLN), for receptor binding preference. A/Chicken/Jiangsu/X1/2004 (X1 H9N2) and A/Puerto Rico/8/1934 (PR8, H1N1) were used as controls for α-2, 3 sialic acid (SA) and α-2, 6 SA, respectively (Figures 5A,B). As described in Figures 5C,D, MA and WT E-Teal/417 mainly bound to α-2, 3 SA, however, MA E-Teal/417 showed stronger binding activity to α-2, 3 SA than WT E-Teal/417. Interestingly, MA and WT GWF-Goose/740 could bind to both α-2, 3 SA and α-2, 6 SA with similar activity (Figures 5E,F).

### Polymerase activity of the MA H6 viruses

**Figure 6:**
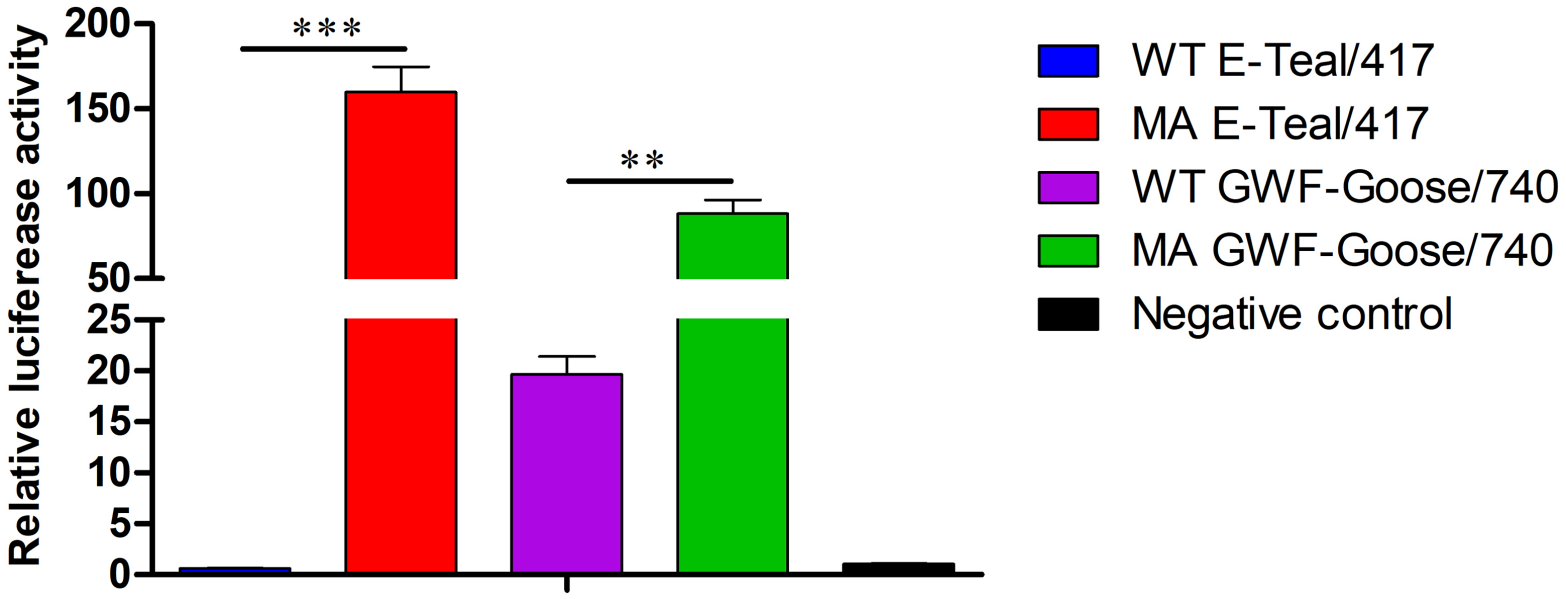
Viral polymerase activity of the MA H6 viruses. The viral polymerase activity of the MA and WT H6 viruses were determined using mini-genome luciferase assays in 293T cells. ^*^, ^**^, and ^***^ indicate *p* values of less than 0.05, 0.01, and 0.001, respectively.

The both MA H6 viruses contained amino acid mutations in RNP polymerase proteins (PB2-T224A/E627K in MA E-Teal/417, PB1-K577E, and PA-T97I/D514E in MA GWF-Goose/740). To assess whether these mutations in MA E-Teal/417 and MA GWF-Goose/740 affect the RNP activity, a mini-genome reporter assay was used for comparing the RNP polymerase activity for the MA and WT H6 viruses in 293 T cells. As shown in Figure 6, the RNP polymerase activity of both MA H6 viruses was significantly higher than that of the corresponding WT viruses. The RNP polymerase activity of MA E-Teal/417 and MA GWF-Goose/740 increased about 200-fold and 4-fold, respectively, when compared to that of the corresponding WT viruses (Figure 6). These data suggest the increased RNP polymerase activity of both MA H6 viruses might contribute to the high pathogenicity of the two mouse adapted H6 viruses.

## Discussion

Avian influenza viruses continue to pose a great challenge for public health, but most studies focus on the H5 and H7 subtype AIVs. In recent years, H6 subtype AIVs have a worldwide distribution, and have been detected in various animal species, including birds, dogs, pigs, and humans (Huang et al., 2010; Shi et al., 2013; Wei et al., 2013; Lin et al., 2015; Sun et al., 2017). Some of the H6 AIVs circulating in poultry in China have enhanced affinity to α-2, 6-linked glycans (Wang et al., 2014). Notably, H6 AIVs could efficiently replicate in mice without pre-adaptation (Li et al., 2021; Wan et al., 2021), and occasionally infected human (Wei et al., 2013). However, the mechanism of H6 AIVs adaptation in mammals remains to be elucidated. In this study, five avirulent H6 subtype AIVs isolated from wild birds were serially passaged in BALB/c mice to evaluate these adaptation in mammalian host. We found that these five H6 AIVs had different potential to be adapted in mice, and only two mouse-adapted H6, designated as MA E-Teal/417 and MA GWF-Goose/740, were efficiently generated through eight passages. It should be noted that although E-Teal/417 with passage 1 to 6 and GWF-Goose/740 with passage 4 to 6 could efficiently replicate in the mice lung during the adaptation, E-Teal/417 and GWF-Goose/740 could not cause significant bodyweight loss before passage 7, which indicated that the high virus titer of H6 AIVs in the mice lung was not enough for inducing high pathogenicity for the mice. We also found that E-Teal/49 could cause significant bodyweight loss in mice at passage 7, whereas this phenotype was reversed at passage 8. Therefore, to confirm the pathogenic phenotype of MA E-Teal/417 and MA GWF-Goose/740, the MLD_50_ of the stock viruses of MA E-Teal/417 and MA GWF-Goose/740 was tested. MLD_50_ of MA E-Teal/417 and MA GWF-Goose/740 was 10^3^ TCID_50_ and 10^3.5^ TCID_50_, respectively (Figure 4), confirming the adapted phenotype of MA E-Teal/417 and MA GWF-Goose/740 in mice.

In comparison with the corresponding WT viruses, genome sequencing and analysis identified mutations of PB2-T224A/E627K, HA-G124R, NA-F167P/Y356H, and M1-M192R in the MA E-Teal/417 (Table 1), and PB1-K577E, PA-T97I/D514E and HA-T276K in MA GWF-Goose/740 (Table 2), which might contribute to the mouse adaptation of the two H6 AIVs. Notably, the same mutation between MA E-Teal/417 and MA GWF-Goose/740 was not identified. This also indicated that different H6 AIV strains with unique gene constellation had different routes to adapt to mammals, and multiple mutations from different gene fragments might response to the host adaptation. Moreover, large sequences analysis revealed that PB2-T224A/E627K, HA-G124R, NA-Y356H in MA E-Teal/417, and PA-T97I, HA-T276K in MA GWF-Goose/740, were also found in some of H6 field isolates, posing the risk for cross-transmission and increased pathogenicity in mammals. Receptor binding assay revealed that MA E-Teal/417 showed stronger binding activity to α-2,3 SA than WT E-Teal/417, whereas MA and WT GWF-Goose/740 could bind to both α-2,3 SA and α-2,6 SA with similar activity (Figures 5E,F), indicating that HA-G124R in the MA E-Teal/417 can increase the HA binding affinity to α-2,3 SA, whereas HA-T276K in MA GWF-Goose/740 can not alter the HA binding profile.

Ribonucleoprotein (RNP) polymerase activity is considered to be one of key factors for the virulence and host adaptation of AIV (Gao et al., 2009; Gabriel et al., 2013; de Graaf and Fouchier, 2014). In this study, five amino acid substitutions, PB2-T224A/E627K, PB1-K577E, and PA-T97I/D514E, were found in the viral polymerase subunits PB2, PB1, and PA. As shown in Figure 6, the polymerase activity of the RNP from the two MA H6 viruses was significantly higher than corresponding WT viruses, which suggested that the two H6 viruses with these mutations in RNP increased virulence in mice. Notably, PB2-E627K and PA-T97I have been reported to be associated with enhanced replication and polymerase activity in mammalian cells, and can increase the virulence in mice (Cheng et al., 2014). The PB1-K577E mutation can increase the pathogenesis of H9N2 in mice (Kamiki et al., 2018). Therefore, we thought that PB2-E627K in MA E-Teal/417, and PB1-K577E and PA-T97I/in MA GWF-Goose/740, play critical roles in the increased pathogenicity of the MA H6 viruses in mice. However, the exact contribution for the increased virulence for each mutation identified in the RNP of the two MA H6 needs further investigation.

In conclusion, our study demonstrates that H6 AIVs from wild birds just need limited mutations (Tables 1, 2) for adaptation in BALB/c mice, indicating the field H6 AIVs with such mutations pose a potential threat to mammal species or public health. Therefore, the continued molecular surveillance of H6 AIVs and the in-depth elucidation of the molecular mechanism for the adaptation of H6 AIVs in mammals are urgently needed.

## Data availability statement

The raw data supporting the conclusions of this article will be made available by the authors, without undue reservation.

## Ethics statement

The animal study was reviewed and approved by Yangzhou University.

## Author contributions

ZW, AQ, and JY conceived and designed the experiments. ZW, JG, JS, YL, TT, WJ, ZZ, ML, WG, and HS performed the experiments. ZW, JG, AQ, and JY analyzed the data and contributed to the writing of the manuscript. All authors contributed to the article and approved the submitted version.

## Funding

This work was support by the National Natural Science Foundation of China (32002262), Basic Research Program of Jiangsu Province (BK20200922) and the Priority Academic Program Development of Jiangsu Higher Education Institutions (PAPD).

## Conflict of interest

JS is employed by Sinopharm Yangzhou VAC Biological Engineering.

The remaining authors declare that the research was conducted in the absence of any commercial or financial relationships that could be construed as a potential conflict of interest.

## Publisher’s note

All claims expressed in this article are solely those of the authors and do not necessarily represent those of their affiliated organizations, or those of the publisher, the editors and the reviewers. Any product that may be evaluated in this article, or claim that may be made by its manufacturer, is not guaranteed or endorsed by the publisher.

## Footnotes

^1^ https://www.fludb.org
